# A review on the formulation and performance of epoxidized vegetable oil-based vitrimer: stoichiometric calculations, curing agent functionalities and catalyst efficiency

**DOI:** 10.1098/rsos.250612

**Published:** 2025-10-15

**Authors:** Chuan Li Lee, Balkis Fatomer A. Bakar, Kit Ling Chin, Luqman Chuah Abdullah

**Affiliations:** ^1^ Institute of Tropical Forestry and Forest Products, Universiti Putra Malaysia, 43400 UPM Serdang, Selangor, Malaysia; ^2^ Bioprocessing and Biomanufacturing Research Complex, Faculty of Biotechnology and Biomolecular Sciences, Universiti Putra Malaysia, 43400 UPM Serdang, Selangor, Malaysia; ^3^ Faculty of Forestry and Environment, Universiti Putra Malaysia, 43400 UPM Serdang, Selangor, Malaysia; ^4^ Department of Chemical and Environmental Engineering, Faculty of Engineering, Universiti Putra Malaysia, 43400 UPM Serdang, Selangor, Malaysia

**Keywords:** vitrimer, stoichiometry ratio, epoxidized vegetable oil, self-healing, formulation

## Abstract

Vitrimers have emerged as an innovative class of polymeric materials that combine the robustness of thermosets with the reprocessability of thermoplastics through dynamic covalent bond exchange. While extensive research has focussed on their mechanical performance and processing conditions, the fundamental role of stoichiometric balance in vitrimer formulations remains underexplored. This review explores the influence of stoichiometry on vitrimer network formation, with a particular emphasis on bio-based epoxy vitrimer systems incorporating epoxidized vegetable oil (EVO). The impact of different epoxy-to-EVO and curing agent-to-EVO ratios on crosslinking density, thermal stability and self-healing characteristics are discussed in detail. The discussion includes the selection of curing agents, namely those with carboxyl (-COOH) and amine (-NH) functional groups, regarding their influence on vitrimer reactivity and recyclability. Notably, findings indicate that EVO-based vitrimer require lower catalyst loading compared to conventional epoxy-based vitrimer, contributing to more sustainable and cost-effective formulations. Understanding the stoichiometric interplay in vitrimer formulations is crucial for optimizing material performance, minimizing catalyst requirements and enhancing long-term durability. Resolving these stoichiometric problems is crucial for the growth of vitrimer applications in biodegradable composites and other high-performance industries.

## 1. Introduction

The quest for high-performance, reprocessable and sustainable materials has prompted significant research into polymeric systems that address the shortcomings of traditional thermosetting and thermoplastic materials. In recognition of their remarkable mechanical strength, thermal stability and chemical resistance, epoxy resins are widely employed in coatings, adhesives and structural composites [1,2]. Nonetheless, their extensively crosslinked structure leaves them permanently infusible and insoluble, resulting in non-recyclability and contributing to material waste, elevated production costs and environmental issues [3,4]. Although thermoplastics provide reprocessability and recyclability, they frequently exhibit insufficient mechanical integrity, temperature resistance and long-term durability essential for rigorous industrial applications, including aerospace, automotive and electronic components [5,6]. The essential trade-off between mechanical durability and recyclability has resulted in the creation of vitrimer materials, which combine the benefits of both thermosets and thermoplastics while ensuring sustainability [7].

Vitrimers are a class of polymers that exhibit reprocessability and self-healing capabilities through dynamic covalent bond exchange, while maintaining stable structural integrity across processing cycles [8]. Opposed to traditional thermosets that establish permanent, irreversible covalent crosslinks, vitrimers have dynamic covalent networks capable of bond rearrangement when subjected to external stimuli like heat or catalysts [9]. This adaptability enables vitrimers to reform, self-repair and undergo reprocessing repeatedly without substantial degradation, rendering them appealing for applications that necessitate prolonged service life, diminished material usage and improved recyclability. Unlike chemically recyclable polymers that necessitate complete depolymerization for reuse, vitrimers can be reprocessed in a solid state, rendering them especially suitable for industrial-scale applications [10].

To fully exploit vitrimer characteristics, researchers have investigated many external processing parameters, including curing temperature, curing duration, and the nature of the curing agent [11–13]. While extended curing times have been associated with higher mechanical strength and prolonged thermal stability, elevated curing temperatures have been shown to accelerate dynamic bond exchange reactions, boost crosslinking density, and improve vitrimer self-healing characteristics [14]. The selection of curing agent significantly influences network development and the kinetics of bond rearrangement. Although these criteria are essential for vitrimer optimization, they do not solely dictate the material’s thermal and mechanical performance. Despite identical curing conditions, vitrimer characteristics such as stress relaxation, reprocessability and structural integrity may demonstrate variability among different formulations. This indicates that a fundamental parameter has not been sufficiently investigated.

In contrast to external curing conditions, which can be altered after formulation, stoichiometric imbalances are inherent to the initial synthesis and cannot be amended post-curing. The stoichiometric ratio of an industrial epoxy/anhydride curing agent ranges from 1 : 0.8 to 1 : 0.9 [15,16]. However, as research studies by [17] reveal, the cured epoxy networks cannot be recycled at such stoichiometric ratios because the quantity of free hydroxyl groups is too low for transesterification. When there is a 1 : 1 equivalence between epoxy and amine groups, no free amines remain, leading to much lower exchange rates compared to systems with free amines. This phenomenon is especially pronounced in *ortho*-substituted vitrimers. The presence of excess amine groups introduces free amine moieties that act as covalently embedded internal catalysts, reducing the activation energy and accelerating the exchange reaction, thereby shortening the relaxation time [18]. Moreover, a study on carbon fibre-reinforced polymer composites with 4-aminophenyl disulfide found that a 1 : 1.2 epoxy/amine ratio preserved a high *T*_g_, strong mechanics and improved reprocessability, showing that such adjustments can boost bond exchange without sacrificing performance [19]. This emphasizes a key weakness in modern vitrimer research, in which the emphasis on processing parameters has replaced the more important influence of stoichiometry on vitrimer properties. In the absence of comprehensive studies on stoichiometry-driven vitrimer behaviour, researchers persist in employing empirical trial-and-error methods, hence obstructing the advancement of predictive models for vitrimer design. Comprehending the impact of stoichiometric changes on vitrimer crosslinking behaviour, thermal stability and mechanical performance is crucial for developing precisely controlled vitrimer networks with reliable durability, recyclability and self-healing efficacy.

The stoichiometric ratio of reactive components plays a fundamental role in determining the performance characteristics of vitrimer systems. While this ratio is important in conventional vitrimer formulations, the integration of bio-based components such as epoxidized vegetable oils (EVO) introduces additional complexities. The unique functional group density and reactivity of EVO differ significantly from typical epoxy resins, which can substantially influence crosslinking efficiency, the kinetics of dynamic bond exchange and ultimately the mechanical, thermal and self-healing properties of the resulting vitrimer [20]. Despite the clear sustainability benefits offered by EVO-derived vitrimers, their effects on thermal stability and mechanical integrity have not been thoroughly investigated, resulting in discrepancies and variability within the existing literature. A more comprehensive understanding of how EVO incorporation impacts vitrimer network formation and dynamic behaviour is therefore essential to address these inconsistencies and to advance the development of robust, high-performance bio-based vitrimer materials.

This review article strives to conduct a study of the impact of stoichiometric ratios on the characteristics of vitrimers, specifically within EVO-based vitrimer formulation. This study aims to create an in-depth and potentially reproducible framework for vitrimer development by prioritizing stoichiometry over external processing conditions as a fundamental design element. This review highlights the importance of EVO-to-epoxy and curing agent-to-epoxy ratios on vitrimer network formation, mechanical integrity and self-healing efficacy, while also examining the effects of various curing agents on bond exchange kinetics and vitrimer stability. Moreover, options for enhancing vitrimer thermal resistance and mechanical durability via meticulous stoichiometric regulation are examined. Rectifying these deficiencies is crucial for advancing vitrimer research from empirical optimization to a predictive, scalable and industrially feasible framework. Gaining a comprehensive understanding of the correlation between stoichiometry and the thermal and mechanical properties of vitrimers would facilitate the creation of advanced vitrimers that are high-performing, durable, environmentally sustainable and economically viable.

## 2. Vitrimers: bridging the gap between thermoplastics and thermosets

The origins of epoxy vitrimer research can be traced back to the early investigations by Dušek and coworkers in the 1970s and 1980s, who explored the role of transesterification reactions in modifying the network structure of epoxy systems. Their seminal work was then contextualized by Williams and coworkers in the field of vitrimer, emphasizing the significance of side reactions in (off)-stoichiometric formulations, a factor often overlooked in contemporary studies [21–23]. However, it was not until the groundbreaking work of Leibler and colleagues in 2011 that the term vitrimer was introduced, describing covalent adaptable networks (CANs) capable of undergoing topology rearrangement via transesterification at elevated temperatures [24–27]. This milestone triggered a surge in research, particularly on epoxy-carboxylic acid systems, focussing on applications such as bio-based coatings, self-healing materials and multi-stimuli–responsive technologies. While transesterification is widely acknowledged as the fundamental mechanism underlying these properties, the specific impact of these reactions on the evolving network architecture which was initially emphasised by Dušek’s early studies and remains an area requiring deeper theoretical and experimental investigation. Other dynamic covalent exchange reactions, including imine and disulfide mechanisms, have attracted considerable interest owing to their reversible bonding under mild conditions, which promotes rapid stress relaxation and efficient self-healing. These properties render them ideal candidates for vitrimers that require reprocessability and self-repair capabilities. For instance, damaged surfaces can often be restored simply by applying heat. Additionally, the ready availability of commercial primary amines and isocyanate monomers offers a flexible foundation for designing a wide range of self-healing and recyclable polyurea-based materials [28,29].

**Figure 1.**
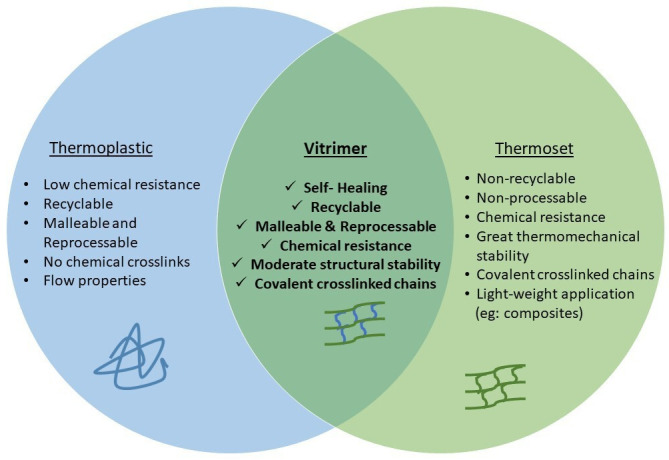
Structural relationship between thermoplastic elastomers, thermoset elastomers and elastic vitrimers.

As illustrated in figure 1, vitrimers bridge the gap between thermoplastics and thermosets by using dynamic covalent adaptable networks. Thermoplastics are characterized by their linear or branched molecular structures with reversible physical interactions, which grant them flexibility, recyclability and reprocessability. However, the absence of permanent crosslinks causes them to have reduced mechanical strength and chemical resistance [30,31]. On the contrary, although the permanent covalent bonds restrict reprocessing and recycling, thermosets exhibit a highly crosslinked network that offers exceptional chemical resistance, mechanical robustness, and thermal stability [32]. Vitrimers occupy an intermediate position by incorporating dynamic covalent crosslinks that allow topological rearrangement without compromising structural integrity. This unique property enables vitrimers to retain the mechanical strength and chemical resistance of thermosets while achieving recyclability, malleability and self-healing properties [33]. As opposed to conventional thermosets, which become non-recyclable and non-processable post-curing, vitrimers can experience stress relaxation and molecular reconfiguration when subjected to external stimuli, such as heat, enabling them to be reshaped, mended and reused. This adaptability makes vitrimers highly promising for applications requiring durability, self-healing efficiency and enhanced sustainability, particularly in coatings, adhesives and structural composites [7,34].

Furthermore, vitrimers enable closed-loop recyclability through controlled heating, facilitating material recovery without extensive degradation. The incorporation of bio-based monomers, such as lignin and rosin derivatives, further enhances the environmental benefits of vitrimers, making them attractive for applications where biodegradability and low environmental impact are desired [24,35]. Recent advances have also demonstrated their potential in high-performance composites, where their unique ability to undergo damage repair and reprocessing significantly extends the lifespan of materials used in aerospace, automotive and construction industries. Despite their promising attributes, several challenges remain in optimizing vitrimer processing conditions, enhancing their mechanical performance and improving large-scale production feasibility. Although vitrimers present promising opportunities for the development of recyclable and reprocessable polymeric materials, they continue to face a significant limitation—dimensional instability and creep under extended use. Commonly referred to as the ‘Achilles’ heel’ of vitrimers, this drawback constrains their practical implementation, as many vitrimer systems exhibit a marked reduction in mechanical performance after only a few reprocessing cycles [36]. The dynamic covalent bonds that facilitate reshaping and self-healing also contribute to time-dependent deformation, thereby undermining their reliability in applications requiring long-term structural stability. Furthermore, the continual evolution of network topology over time, driven by bond exchange reactions, adds complexity to predicting and maintaining mechanical integrity during prolonged service [37]. Thus, the fine-tuning of dynamic bond chemistry is critical to balancing reprocessability with mechanical integrity, ensuring that vitrimers maintain their advantageous properties across various applications. Ongoing research continues to explore novel formulations, alternative dynamic covalent bonding mechanisms and new applications, paving the way for vitrimers to become a transformative material class in sustainable polymer science.

### 2.1. Dynamic covalent bond exchange mechanisms in vitrimers

Building upon the structural advantages and recyclability of vitrimers, the key to their dynamic behaviour lies in the incorporation of dynamic covalent bond exchange mechanisms. These mechanisms govern the vitrimer’s ability to undergo stress relaxation, reprocessing and self-healing while maintaining the integrity of the crosslinked network. Several dynamic bond exchange mechanisms have been employed in vitrimer synthesis, including transesterification, disulfide exchange, imine metathesis, boronic ester exchange, dynamic urea bonding and siloxane exchange [38,39]. Each of these mechanisms influences the vitrimer’s thermal, mechanical and reprocessability properties, allowing for tailored functionality depending on the intended application. Table 1 summarizes key characteristics of various covalent adaptive network mechanisms, including the chemical reactions involved, catalysts mechanical and thermal properties, advantages and limitations.

**Table 1.** Comparison of dynamic covalent bond exchange mechanisms and their effects on vitrimers’s properties.

mechanism	chemical equation	catalyst/trigger	mechanical and thermal properties	advantages	limitations	reference
transesterification	R-COO-R’+R’-OH ⇌ R-COO-R’+R’-OH	zinc salts	high elasticity, moderate thermal stability	good reprocessability, maintains mechanical integrity	requires catalyst, potential moisture sensitivity	[34,40]
disulfide exchange	R-S-S-R+2R’SH ⇌ 2R-SH+R’-S-S-R’	heat, UV light, oxidizing agents	enhanced toughness, self-healing capabilities	enables recyclability, self-healing properties	reduced thermal stability, sensitivity to oxidation, complex process, and involves several mechanisms that also depend on the used conditions and substitution patterns of the disulfides	[41–43]
imine bond formation	R-CHO+R’-NH₂ ⇌ R-CH = N R’+H₂O	lewis or brønsted acids	good self-healing efficiency, moderate mechanical strength	dynamic bond exchange under mild conditions	moisture sensitivity, potential hydrolysis	[44–46]
boronic ester exchange	R-B(OR’)₂+R’-OH ⇌ R-B(OR’)(OR’)+R’-OH	water, elevated temperatures	high reprocessability, moderate mechanical properties	reversible bond formation, tunable properties	limited chemical resistance, moisture sensitivity	[47–49]
dynamic urea bonding	R-NH-CO-NH-R’+H₂O ⇌ R-NH₂+R’-NHCOOH	high temperatures (>150°C), moisture	high thermal stability, good mechanical strength	enhanced chemical resistance, thermal stability	requires high processing temperatures	[48,50]
siloxane exchange	R-Si-O-Si-R’+H₂O ⇌ 2R-Si-OH	moisture, acid/base catalysts	excellent thermal stability, flexibility-highly sort after properties such as elastic deformation and excellent elongation under tensile loads	high reprocessability, stability under harsh conditions, repeatedly perform without failure	lower mechanical strength compared to carbon-based networks- low tear strength	[51–53]

Additionally, a deep understanding of dynamic covalent bond exchange is essential to tailor vitrimer properties such as thermal stability, mechanical strength and reprocessability. These exchanges occur within CANs, which fall into two categories: associative and dissociative. Each type follows a different exchange mechanism and exhibits distinct material behaviours, as summarized in table 2. Dissociative CANs, first introduced in 2002, have contributed to the development of chemistries enhancing reprocessability and self-healing. Although some dissociative CANs display vitrimer-like Arrhenius viscosity behaviour above the glass transition temperature (*T*_g_), their bond exchange mechanism fundamentally differs. In these systems, bond cleavage occurs before new bond formation, temporarily reducing crosslink density and potentially compromising mechanical and thermal stability during processing. Conversely, associative CANs are commonly referred to as vitrimers which maintain stable crosslink density through an addition–elimination exchange mechanism. As reported by Leibler and coworkers in 2011, such networks with associative ester bond exchanges exhibit silica- or glass-like malleable behaviour at elevated temperatures while preserving mechanical strength, thermal stability and solvent resistance.

**Table 2.** Associative versus dissociative CANs.

aspect	associative CANs	dissociative CANs	reference
bond exchange mechanism	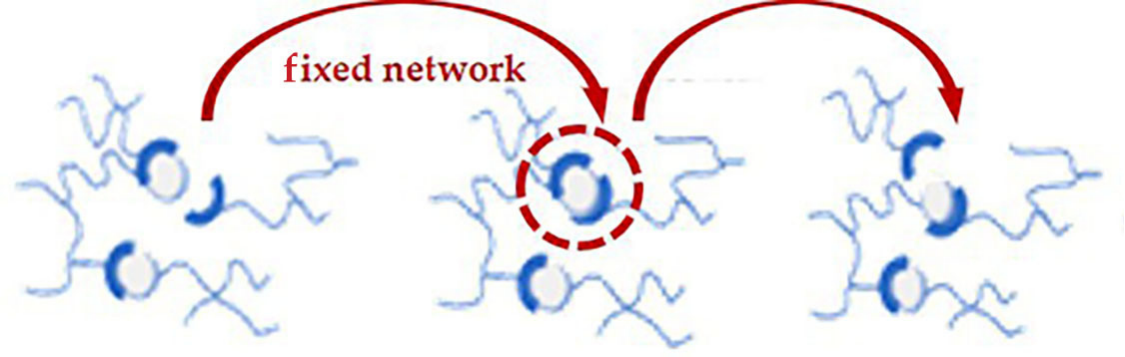	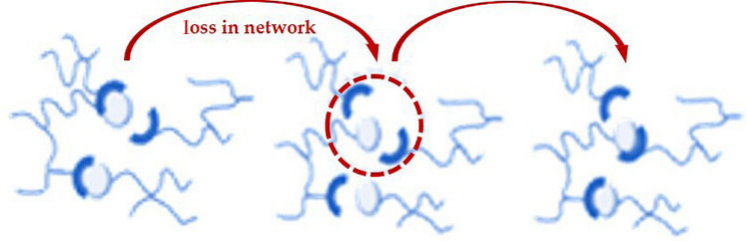	
description	a new bond forms before the original bond breaks, following an addition–elimination pathway. This preserves the overall network connectivity during the exchange	the original bond breaks before a new bond forms, following an elimination–addition pathway. This leads to a temporary loss of network integrity during the exchange	[54]
crosslink density during exchange	remains constant, preserving mechanical properties during processing	temporarily decreases, which can affect mechanical stability during processing	[54]
**typical chemistries**			
transesterification	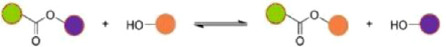	-	[55]
vinylogous urethane exchanges	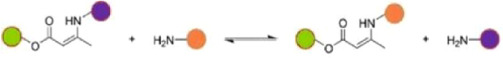	-	
boronic esters exchanges	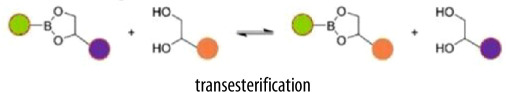	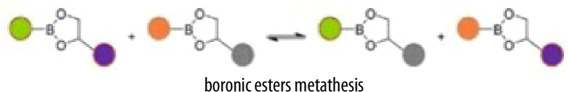	
urethane-based exchanges	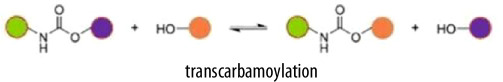	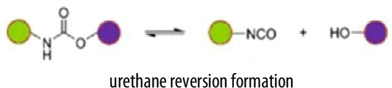	
hydroxyurethane- based exchanges	transcarbamoylation	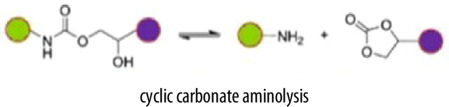	
imine-based exchanges	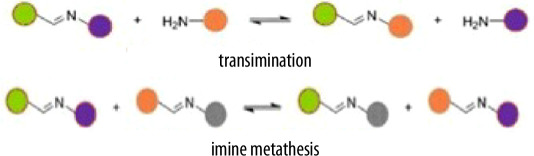	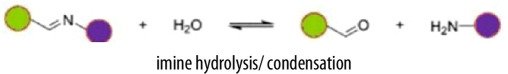	
disulfide exchange	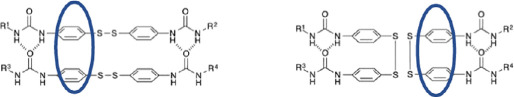 disulfide metathesis	-	
thermal stability	high thermal stability with associative ester bonds enabling network integrity and glass-like malleability at elevated temperatures	low thermal resistance owing to the significant drop of crosslink density upon application of heat	[56]
reprocessability	demonstrates excellent reprocessability with consistent crosslink density, regardless of stimuli conditions	exhibits good reprocessability alongside self-healing capabilities	[56]

As shown in tables 1 and 2, transesterification, with its balanced mechanical performance and processability, stands out among the dynamic covalent methods as the most effective for vitrimer applications. Compared to disulfide exchange, which enhances self-healing but suffers from oxidation sensitivity, transesterification maintains superior stability while enabling recyclability. However, conventional transesterification often relies on non-renewable monomers, highlighting the need for sustainable alternatives [57]. Recent studies have demonstrated the feasibility of bio-based transesterification vitrimers, using renewable monomers such as epoxidized soybean oil (ESO) and citric acid (CA), which exhibit self-healing and recyclability without requiring additional catalysts [58,59]. These advancements reinforce the potential of bio-based vitrimers to reduce environmental impact while maintaining the dynamic covalent network properties that facilitate reprocessing and durability. Consequently, the development of bio-based vitrimers has attracted growing interest since they provide an environmentally friendly substitute without compromising functional performance.

### 2.2. Bio-based vitrimers: a sustainable approach

Bio-based vitrimers are a class of polymeric materials derived from renewable resources that incorporate dynamic covalent bonds, enabling recyclability, self-healing and reprocessability. By using bio-based precursors such as vegetable oils [60,61], lignin [32,62], natural rubber [63,64], vanillin [65,66], rosin [67,68] and cardanol [68,69], these materials provide a sustainable alternative to traditional petroleum-based polymers.

#### 2.2.1. Classification of bio-based vitrimers: fully and partially bio-based vitrimer

**Figure 2.**
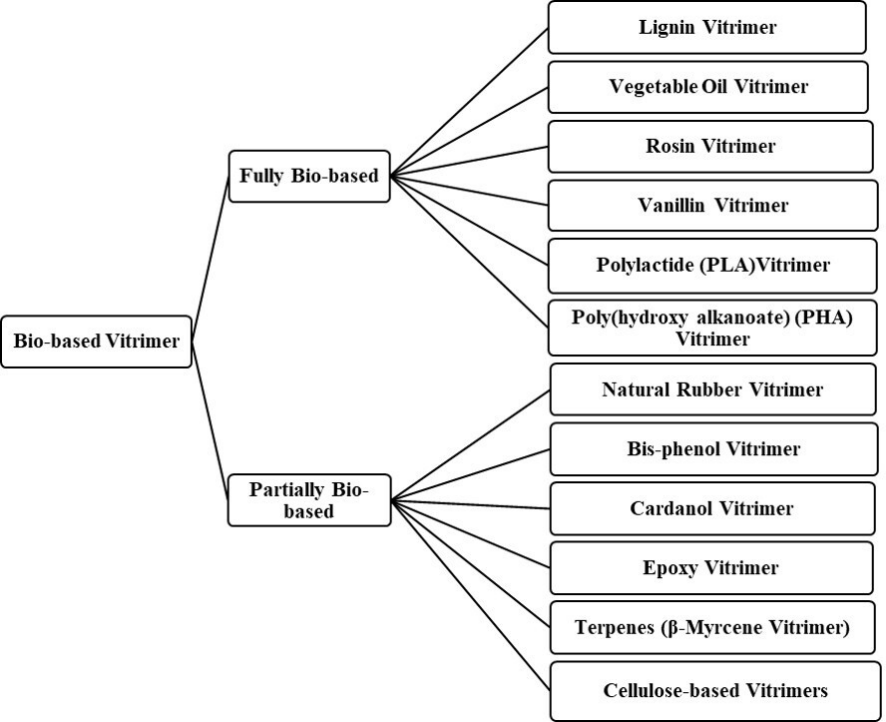
Classification of bio-based vitrimers (adapted from [24]).

Based on the proportion of renewable components in their formulation as indicated in figure 2, bio-based vitrimers can be categorized as either fully or partially bio-based. Fully bio-based vitrimers entirely rely on renewable raw materials, making them highly sustainable with low environmental impact. Conversely, partially bio-based vitrimers still incorporate synthetic fractions, such as petroleum-based monomers or curing agents, to enhance mechanical strength, thermal resistance, or processing stability [55].

#### 2.2.2. Performance and applications of bio-based vitrimers

Bio-based epoxy vitrimers exemplify the successful integration of rigidity and recyclability. These materials can be reprocessed through mechanical hot pressing, enhancing thermal resistance and mechanical strength while maintaining recyclability. Their degradability in alkaline solutions renders them environmentally friendly replacements for commercial bisphenol-A epoxy resins used in plastic manufacture [70]. Likewise, natural rubber vitrimer-like elastometers are environmentally benign substitutes for traditional rubber vulcanizates since they show repairable, weldable, recyclable and reprocessable qualities. These materials, which line up with the ideas of a circular economy, provide increased durability while cutting rubber waste [55]. The development of bio-based vitrimers aligns with the growing demand for eco-friendly polymeric materials, offering both functional advantages and environmental benefits. Their ability to extend the lifespan and enhance the performance of traditional thermosets makes them promising candidates for various industrial applications [24]. As shown in figure 2, vanillin, a biomass-derived compound, is often regarded as a fully bio-based precursor; however, its chemically modified derivatives, such as vanillin methacrylate (VMA), incorporate synthetic elements that make them only partially bio-based [71,72]. For instance, imine-based vitrimers synthesized from diimine-dimethacrylate monomers are prepared by condensing VMA with hexamethylenediamine [73]. While these systems include renewable components, the overall bio-based content depends on all constituents in the polymer network, highlighting the importance of careful assessment in determining their sustainability and bio-based classification.

Moreover, cross-linked elastomers are typically stretchable but lack recyclability and biodegradability. However, certain chemical motifs, such as those in medium-chain-length polyhydroxyalkanoates, offer intrinsic ductility and biodegradability, making them promising bio-based elastomers [37]. Likewise, cellulose-based vitrimer composites formed via *in situ* polycarbonate network generation within porous cellulose fibre paper using Ti(IV)-catalysed *trans*-carbonation demonstrate enhanced mechanical strength. The interplay of hydrogen bonding and dynamic covalent cross-links enables shape memory, self-healing and reprocessability under mild conditions [74]. These techniques not only encourage the use of renewable materials and dynamic covalent chemistry but also offers a scalable and industrially viable pathway towards next-generation vitrimer composites compatible with circular economy aims.

#### 2.2.3. Challenges and limitations of partially bio-based vitrimers

Although partially bio-based vitrimers provide a compromise between renewability and performance, they have distinct drawbacks relative to fully bio-based alternatives. The incorporation of petroleum-derived monomers or crosslinkers reduces their overall sustainability and biodegradability. The reliance on synthetic components causes end-of-life degradability less environmentally beneficial and can impede the material’s complete reintegration into natural cycles [75]. Furthermore, the limited renewable content in partially bio-based vitrimers results in a higher carbon footprint compared to fully bio-based counterparts. Regulatory frameworks and sustainability goals increasingly favour materials with higher bio-based content, potentially limiting the applicability of partially bio-based vitrimers in stringent eco-friendly policies. The potential toxicity of synthetic additives is another concern. Some synthetic crosslinkers and curing chemicals employed in partially bio-based vitrimers could produce harmful byproducts, therefore influencing environmental impact and human safety. Additional purification steps may be required to meet biodegradability and non-toxicity standards. Recyclability constraints also arise owing to the integration of non-renewable fractions, which can affect the efficiency of the dynamic covalent bond exchange mechanisms. Certain synthetic additives may limit multiple recycling cycles, leading to material degradation over time Lastly, trade-offs in mechanical performance are readily apparent. Although incorporating bio-based precursors enhances sustainability, these materials often exhibit inherent limitations such as reduced mechanical strength and thermal resistance. Such drawbacks necessitate the incorporation of synthetic reinforcements, especially in high-performance applications like automotive coatings, electronics and others. A significant challenge stems from the hydrophobic nature of bio-based oils, which complicates their integration into waterborne coatings including water-soluble resins, emulsions, dispersions, latexes and water-reducible resins [76]. These coatings require compatibility with aqueous environments, and this incompatibility adversely affects coating stability, mechanical integrity and thermal performance, thereby presenting considerable obstacles in balancing sustainability with the demanding requirements of advanced applications.

The environmental impact of bio-based and synthetic polymers is closely linked to their carbon footprint, which depends on both the carbon embedded in their molecular structure and the emissions generated throughout production, use and disposal. Fully bio-based materials generally have an inherent carbon footprint close to zero in terms of their chemical structure, as the carbon originates from renewable biomass [77,78]. This carbon can be considered carbon-neutral since it was recently captured from the atmosphere during biomass growth. By contrast, petrochemical-based materials contain stored fossil carbon, which directly contributes to long-term carbon emissions. The process carbon footprint may accounting for energy consumption, raw material sourcing, and manufacturing efficiency—is inherently non-zero and varies based on specific production methods [79,80]. For economic viability, fully bio-based raw materials should avoid competing with food supply by using agricultural waste or non-food crops [75]. Studies on life cycle assessments show that fully bio-based vitrimers have a lower overall environmental impact than partially bio-based ones, thanks to their exclusive use of renewable biomass and better biodegradability [81]. Despite cost variations, fully bio-based materials generally offer superior sustainability. Moreover, synthetic polymers which commonly present in partially bio-based vitrimers might make up approximately 11% of municipal solid waste by mass but occupy disproportionate landfill volume owing to their low density. Unlike many other wastes, these synthetic polymers are not biodegradable and persist indefinitely in landfills, exacerbating environmental concerns [81].

## 3. Epoxidized vegetable oil-based vitrimers: enhancing sustainability and performance

Owing to their remarkable mechanical strength, thermal stability and chemical resistance, epoxy-based vitrimers are among the most extensively investigated bio-based vitrimer formulation. Epoxy resins, particularly those derived from diglycidyl ether of bisphenol-A (DGEBA), have been extensively used in coatings, adhesives, composites and electronic materials owing to their superior durability and resistance to environmental degradation [82,83]. However, conventional epoxy-based vitrimers face several drawbacks, including brittleness, limited flexibility and high dependence on petrochemical-based monomers, which restrict their sustainability and reprocessability [50,84]. To further enhance the sustainability of epoxy vitrimers, researchers have explored bio-based alternatives, particularly vegetable oils, as partial replacements for DGEBA. EVO, such as ESO [85,86], epoxidized linseed oil (ELSO) [84,87], epoxidized castor oil [88,89] and others provide flexibility, improved toughness and enhanced self-healing properties into epoxy vitrimer networks. These bio-based oils contain reactive functional groups that can participate in crosslinking reactions, modifying the vitrimer’s mechanical properties and reprocessability [90,91].

Epoxy resins typically have high viscosity, which limits their processability [92]. Adding smaller, less viscous curing agent molecules helps lower this viscosity and improves handling during processing. EVOs serve as reactive diluents that can partially replace epoxy resin, further reducing viscosity while chemically bonding into the network. According to the research of Ozgul & Ozkul [93], incorporating 20% ESO modifies DGEBA and its rheological properties. This replacement not only simplifies processing but also influences curing behaviour and the final material properties. Therefore, precise control of the stoichiometric ratio between resin, curing agent and EVO is essential to maintain mechanical strength and thermal stability. The study by Czub [94] also further showed that EVO used at concentrations between 20 and 60 wt%, can reduce curing temperatures without compromising cure completeness. In addition, EVO-based systems improve flexibility, elastic recovery, water resistance and chemical durability. When combined with bio-based crosslinkers, EVO helps reduce the brittleness often seen in fully bio-based epoxies. However, the effect of EVO on mechanical properties can vary, as some studies report higher strength and deformation, while others note a lower modulus and ultimate strength but greater ductility [95,96]. This variability highlights the importance of selecting suitable diluents, with the epoxidation process used to produce EVO playing a key role in determining its reactivity and overall performance.

Epoxidation which converts double bonds into reactive oxirane groups is a key modification method to produce the EVO. As summarized in table 3, the fatty acid composition of vegetable oils, particularly the types and proportions of unsaturated acids such as oleic, linoleic and linolenic acids plays a crucial role in determining their epoxidation potential, and the properties of the resulting materials such as oils rich in polyunsaturated linolenic acid, like linseed oil, offer multiple reactive sites for epoxidation, producing densely crosslinked networks with superior thermal stability and mechanical strength [146]. Castor oil, characterized by ricinoleic acid containing both a double bond and hydroxyl group, exhibits enhanced reactivity and imparts flexibility and toughness to vitrimer networks [57]. Soybean and sunflower oils, high in linoleic acid, provide moderate unsaturation that balances rigidity and elasticity, making them suitable for reprocessable materials [147]. These variations in fatty acid profiles significantly influence the thermal, mechanical and recyclability properties of bio-based epoxy vitrimer, enabling tailored design for sustainability and performance. As shown in table 3, recent advances include the use of perpropionic acid combined with reusable solid acid catalysts like Amberlite IR-120, which enhance reaction efficiency, selectivity and thermal stability, providing safer and more effective epoxidation routes [147]. These improvements are particularly important for developing EVO-based vitrimer with optimized crosslink density and dynamic bond exchange capabilities, thereby advancing sustainable, high-performance and reprocessable polymer networks.

**Table 3.** Epoxidation of vegetable oils for sustainable material applications.

substrate	structure	fatty acid	precursor	catalyst	conditions	product/result	reference
canola oil	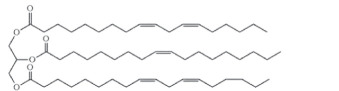	61% oleic acid, 21% linoleic acid, 11% alpha-linolenic acid, 4% palmitic acid, 2% stearic acid 1% other [97]	peroxy-acetic acid	amberlite IR120H	65°C	90% conversion	[98]
			H₂O₂	sulfated-SnO₂	0.5 h	high oxidative stability, viscosity, and lubricity	[99]
			percarboxylic acid	H₂SO₄	65°C, 2% H^+^	81% ethylene conversion	[100,101]
			acetic acid, H₂O₂	amberlite IR−120	55−65–75°C, 2.5−4−5.5 h	95% at 75°C and 2.5 h	[102]
castor oil	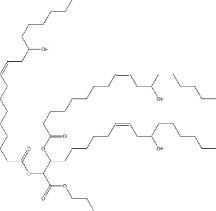	89.5% ricinoleic acid, 4.2% linoleic acid, 3% oleic acid, 1% stearic acid, 1% palmitic acid, 0.7% dihydroxystearic acid, 0.3% linolenic acid, 0.3% eicosenoic acid [103]	peracetic acid/performic acid	amberlite IR−120	Ea = 48.2/35.4 kJ mol^−1^	peracetic / performic acid epoxidation	[104]
			acetic acid+H₂O₂	amberlite	323 K, 8 h	pseudohomogeneous model correction	[105]
corn oil	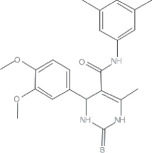	11% palmitic acid, 2% stearic acid, 28% oleic acid, 58% linolenic acid, 1% alpha linolenic acid [106]	stearic acid, H₂O₂	novozym 435	35°C, 10 h, 28% acid	6% epoxy oxygen content, 88.2% relative conversion to oxirane	[107]
			acetic acid, H₂O₂	Acidic ion exchange resin modified with Zn	75°C, 600 rpm, 5.5 h	6.40 wt% of epoxy oxygen content, 87.67% relative conversion to oxirane	[108]
cotton seed oil	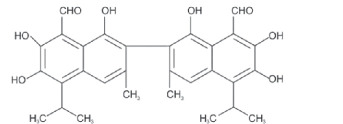	23% palmitic acid, 18% oleic acid, 54% linoleic acid [109]	acetic acid, H₂O₂	sulfuric acid	5 h, 20−27°C	35% yield	[110]
			peracetic acid	HCl, HNO₃, H₂SO₄	60°C	78% conversion	[111]
grape seed oil	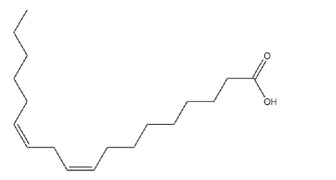	11.85% palmitic acid, 0.68% palmitoliec acid, 5.75% stearic acid, 25.84% oleic acid, 55.25% linoliec acid, 0.40% arachidonic acid, 0.23% gadoliec acid [112]	acetic acid, H₂O₂	H₂SO₄	50−60°C, 1−6 h	greatest epoxide yield at 90°C, 1 h	[113]
			CO₂, H₂O₂	Phase transfer catalyst	40°C, 150 bars, 15 h	8.31% yield	[114]
jatropha oil	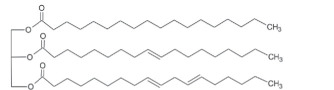	1.1% myristic acid, 15.31% palmitic acid, 0.52% palmitoleic acid, 7.1% stearic acid, 36.72% oleic acid, 39.05% linoleic acid, 0.20% arachidic acid [115]	acetic acid, formic acid, H₂O₂	H₂SO₄	60°C, 5 h	catalyst efficiency	[116]
			formic acid, H_2_O_2_		60°C, 4 h (reaction)	ideal plasticizer Polylactic Acid (PLA)	[117]
			*in-situ,* formic acid, H₂O₂,	Not Reported	60°C, 5 h 1500 rpm	improved thermal decomposition range	[118]
			performic acid	Not Reported	45°C, 2 h	70% yield, 80.4% conversion	[119]
linseed oil	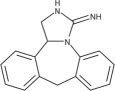	56.6% linolenic acid, 15.3% linoleic acid, 19.1% oleic acid [120]	glacial acetic acid+H₂O₂: forms peracetic acid	Seralite (strong resin cation −120) : solid acid catalyst (25 wt%)	60°)C, 5 h	sustainable bio-based printing inks	[121]
			performic acid, peracetic acid, H₂O₂	alumina	65, 75, and 85°C	direct epoxidation reliability	[122]
			percarboxylic acid	amberlite IR−120	333−358 K, 1−17 h	linolenic acid efficiency	[123]
palm oil	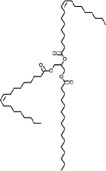	0.2% caproic acid, 3.3% caprylic acid, 3.5% capric acid, 47.8% lauric acid, 16.3% myristic acid, 8.5% palmitic acid, 2.4% stearic acid, 15.4% oleic acid, 39.05% linoleic acid, 0.1% arachidic acid [124]	H₂O₂	formic or acetic acid	60°C, 120 min	as a reactant for bio-based epoxy resins	[125]
			—	Not Reported	55°C, 150 min	49.82% yield, potential for biolubricant	[126]
			acetic acid, H₂O₂	H₂SO₄	70°C, 3 h	11.36% yield	[127]
			formic, H₂O₂	—	60°C, 4 h	self-healing performance	[128]
			peroxoformic acid	Ti–Si 0.5	60°C, 5 h, 640 rpm	84% yield	[129]
			—	H₂SO₄, HCl, HNO₃	55°C, 200 rpm, 20 min	85.6% relative conversion oxirane	[130]
waste palm kernel oil		0.72% oleic acid, 9.76% nonadecylic acid [131]	formic or acetic acid	—	stir speed (300 rpm)	88% yield	[132]
			peracetic acid	H₂SO₄	60°C, 4 h	56.8% yield	[133]
soybean oil	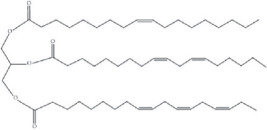	7−10% palmitic acid, 2−5% stearic acid, 1−3% arachidic acid, 22−30% oleic acid 50−60% linoleic acid , 5−9% linolenic acid [134]	acetic acid, H₂O₂	H₂SO₄	60°C, 600 rpm, in slug-flow millireactor	82% yield	[135]
			peracetic acid	H₂SO₄	338 K	higher epoxy yield and selectivity	[136]
			H₂O₂	Titanosilicate TS−1 zeolite/Cd	NR	eco-friendly, non-toxic	[137]
			formic acid, H₂O₂,	phosphoric acid	60−75°C, 8−10 h	biphasic model improvement	[138]
sunflower oil	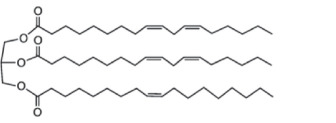	12.44% palmitic acid, 4.51% stearic acid, 23.30% oleic acid, 55.03% linoleic acid, 4.74% linolenic acid [139]	formic acid, H₂O₂,	zeolite	70°C (200, 300, or 400) rpm	52% yield	[140]
			acetic acid, H₂O₂	amberlite resin, and toluene	several hours at 70°C	flame-retardants	[141]
waste sunflower oil		0.8% oleic acid, 0.36% palmitic acid, 0.10% linoleic acid, 0.26% erucid acid, 0.20% caprylic acid [131]	carboxylic acid, H₂O₂	Not Reported	4 h	82.91% yield	[142]
tung oil	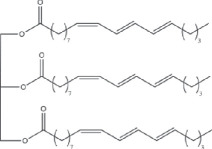	76.5% a-eleostearic acid, 2.4% b-eleostearic acid, 2.7% palmitic acid, 6% oleic acid, 1.3% linolenic acid, 0.2% arachidic acid, 8.2% linoleic acid, 2.6% stearic acid [143]	acetic acid, H₂O₂	H₂SO₄	50−60°C, 500 rpm, 4 h	kinetic model at optimum conditions	[144]
			1.5% catalyst, 1.6 H₂O₂	4 h	kinetic model proposed	48.94% conversion	[145]

Despite the promising advantages of EVO-based vitrimers, several critical factors influence their overall performance and applicability. The compatibility between rigid DGEBA structures and flexible bio-based precursors plays a crucial role in achieving a uniform network, as phase separation can lead to inconsistencies in mechanical properties. Additionally, optimizing the ratio of vegetable oil to epoxy resin, selecting appropriate curing agents and fine-tuning catalyst formulations are essential to maximize crosslinking efficiency and ensure thermal and mechanical stability. Recent studies have shown that the crosslinking kinetics and reprocessability of bio-based vitrimers was influenced by the choice of catalysts, especially zinc-based and organic variations [148]. By addressing these important aspects, EVO-based epoxy vitrimers can be further developed into high-performance, sustainable polymeric materials for coatings, adhesives and structural composites.

### 3.1. Influence of stoichiometric ratio on epoxidized vegetable oil-based vitrimer properties

The stoichiometric ratio of reactive functional groups in vitrimer networks is crucial in determining the mechanical characteristics, reprocessability and overall stability. A well-structured crosslinked network is ensured by reaching an ideal equilibrium between functional groups, such as epoxy and carboxyl groups, therefore reducing undesired side reactions and increasing the efficacy of dynamic covalent bond interactions [23]. A crucial yet sometimes neglected aspect of vitrimer design is the accessibility and spatial configuration of reactive centres or crosslinks. In vitrimer networks, the associative bond-exchange process necessitates the contact of polymer chain segments prior to the exchange of a crosslink. Thus, stoichiometry significantly influences vitrimer behaviour, especially regarding processability and stress relaxation [7,16].

Precise control of the epoxy-to-carboxyl molar ratio is crucial for attaining an ideal crosslinked architecture while facilitating effective dynamic covalent bond exchange. In epoxy resin formulation, cured using anhydrides as the curing agent, the epoxy-to-anhydride ratio directly affects the availability of hydroxyl groups essential for transesterification reactions [149,150]. A 1 : 1 (epoxy : anhydride) ratio generally ensures a full reaction between functional groups. Adjusting the curing agent ratio regulates the availability of epoxy groups, hence affecting the vitrimer characteristics. Off-stoichiometric formulations with excess epoxy (epoxy-to-anhydride ratio greater than 1) have been explored to improve bond exchange efficiency. Excess hydroxyl groups enhance transesterification, hence increasing stress relaxation properties and vitrimer recyclability [151]. Consequently, modifying the curing agent ratio is a crucial approach for modifying vitrimer characteristics, especially for applications necessitating improved temperature stability and dynamic bond interchange. Beyond the epoxy-to-curing agent ratio, bio-based epoxy monomers have also been incorporated to enhance vitrimer sustainability. A notable example is epoxidized cardanol glycidyl ether, derived from cashew nut shell liquid, in which variations in the carboxyl-to-epoxy ratio directly affect *T*_g_, Young’s modulus and recyclability [152,153]. Such stoichiometric adjustments influence not only mechanical integrity and self-healing capability but also the extent of dynamic covalent bond exchange, which is critical for recyclability.

### 3.2. Effect of epoxidized vegetable oil-to-diglycidyl ether of bisphenol-A ratio on the thermal and mechanical properties of vitrimers

The integration of EVO into vitrimer networks has been extensively studied to improve sustainability and flexibility in polymer materials [154,155]. However, their lower epoxide functionality often results in reduced crosslinking density, which can adversely affect mechanical strength and thermal stability [156]. Complete substitution of conventional epoxy resins with EVO is therefore usually impractical as it reduces vital vitrimer characteristics including reprocessability and self-healing efficiency. Research on partial substitution techniques has concentrated on optimizing the EVO-to-epoxy ratio to balance mechanical performance with recyclability in order to meet these constraints [156]. Studies have shown that moderate EVO incorporation can improve flexibility and reduce brittleness, but excessive EVO content leads to lower *T*_g_ and decreased Young’s modulus [157]. Adjusting the EVO-to-epoxy ratio is therefore critical to maintaining the advantageous properties of traditional epoxy resins while incorporating the sustainability benefits of bio-based components. To further illustrate the influence of stoichiometry in EVO-based vitrimer networks, table 4 presents a comparative analysis of different types of EVO blended with DGEBA, highlighting their effects on curing performance and thermomechanical properties. This comparison demonstrates how varying the stoichiometric ratio can significantly impact crosslinking density, thermal stability and mechanical behaviour.

**Table 4.** The effect of EVO-to-DGEBA ratio in vitrimer formulation.

types of EVO	properties of EVO	curing condition	stoichiometric ratio	thermal properties		mechanical properties		reference
epoxidized soybean oil (ESO) *ESO, EEW = 241 g eq^−1^; average molecular weight = 940 Da; DGEBA, EEW: 185 g mol^−1^	functions as a toughener, plasticizer, and diluent by reducing cross-linking density and internal stress in epoxy resins. Requires epoxidation as soybean oil lacks epoxide groups	methyltetra-hydrophthalic anhydride, 130°C for 1 h and then 190°C for 3 h	ESO/DGEBA: 0/100	*T*_g_,(°C)	108	compressive modulus (GPa)	≈2.0	[158]
			ESO/DGEBA: 20/80		102		≈2.0	
			ESO/DGEBA: 40/60		97		≈1.7	
			ESO/DGEBA: 60/40		85		≈1.3	
			ESO/DGEBA: 80/20		72		≈1.0	
			ESO/DGEBA: 100/0		57		≈0.8	
		ethylene diamine, cure for 1 h at 105°C and post cured for 1 h at 150°C in an oven	ESO/DGEBA: 0/100	char residue(%)	12.1	—		[159]
			ESO/DGEBA: 15/85		12.7			
			ESO/DGEBA: 30/70		13.8			
epoxidized unripe palm oil (ERPO)	enhances flexibility, toughness, impact resistance, and thermal stability. Moderate oxirane content enables dynamic covalent bond exchange	cycloaliphatic amine	ERPO/DGEBA: 0/100	—		cross-cut tape test adhesion: average per cent area removed (%)	0	[160]
			ERPO/DGEBA: 10/90				14.33	
			ERPO/DGEBA: 20/80				4.33	
			ERPO/DGEBA: 30/70				1.0	
epoxidized uused cooking oil (ECKO)	lower viscosity and varied fatty acid composition affect curing and mechanical properties	cycloaliphatic amine	ECKO/DGEBA: 0/100	—			0	
			ECKO/DGEBA: 10/90				78.33	
			ECKO/DGEBA: 20/80				80.0	
			ECKO/DGEBA: 30/70				65.0	
epoxidized castor oil (ECO) *ECO: *M*_W_ ≈975 g/mol, OCC: ≈6.5%; DGEBA: EEW: 183−189 g/eq	exhibits superior durability, low toxicity, and multifunctionality, making it an effective toughener. However, its high cost and limited availability restrict large-scale use	triethylenetetramine, Initial curing was done at room temperature for 24 h and post curing was done at 100°C for 1 h, 120°C for 2 h, and 130°C for 1 h	ECKO/DGEBA: 0/100	char residue (%)	4.3	tensile strength (MPa)	70.18 ± 8	[161]
			ECKO/DGEBA: 10/90		1.6		50.79 ± 6	
			ECKO/DGEBA: 20/80		1.4		54.22 ± 3	
			ECKO/DGEBA: 30/70		0.43		42.41 ± 4	
			ECKO/DGEBA: 50/50		0.45		18.26 ± 2	
epoxidized waste frying sunflower oil (ESFO)	sustainable and cost-effective, but variable composition and potential impurities affect performance	glutaric anhydride, 180°C for 4 h	ESFO/DGEBA: 20/80 ESFO/DGEBA: 40/60 ESFO/DGEBA: 60/40 ESFO/DGEBA: 80/20	—		mechanical properties (Young’s modulus, MPa)	1	[162]
							2	
							2.5	
							2.8	

*EEW = Epoxy Equivalent Weight*

OOC = Oxirane Oxygen Content

The incorporation of EVO, such as ESO, epoxidized palm oil (EPO) and castor oil (ECO), into vitrimer formulations presents both benefits and challenges. However, with the great crosslinking density which offers outstanding tensile strength, stiffness and thermal resistance, DGEBA-based resins are extensively employed in thermosetting applications. Still, some natural constraints limit their relevance. Thus, the ideal balance between DGEBA and EVO is essential to maintain the desired mechanical strength, thermal stability and curing efficiency. As the proportion of EVO increases, a reduction in crosslinking density occurs because of their lower epoxide functionality compared to DGEBA. This reduction leads to a decrease in *T*_g_ value, indicating enhanced molecular mobility and improved flexibility (refer to table 4). However, excessive EVO content results in significant thermal softening, reducing the vitrimer’s ability to maintain structural integrity at elevated temperatures [163].

The molecular weight and oxirane number of EVO significantly influence the properties of vitrimers. A higher oxirane number, indicating increased epoxide functionality, enhances the resin’s crosslinking potential, leading to improved mechanical strength and thermal stability. For instance, ELSO with a high oxirane number, has been shown to produce polymers with higher *T*_g_ value compared to those derived from ESO [164]. Conversely, EVO with higher molecular weights, such as ECO, can improve the toughness and flexibility of the resulting vitrimers. However, their bulkier structures may introduce steric hindrance, potentially reducing crosslinking efficiency and leading to lower *T*_g_ and decreased thermal stability [165]. Therefore, the molecular weight of EVO significantly influences vitrimer properties, offering tunable flexibility and dynamic bonding, unlike the high crosslinking but brittle nature of DGEBA.

Adhesion performance, assessed through cross-cut tape tests, shows that moderate EVO levels improve interfacial adhesion, probably owing to enhanced polymer chain mobility and surface wettability. However, excessive EVO content weakens adhesion strength, leading to greater material detachment. Char residue analysis further indicates that higher EVO content results in less carbonaceous residue during pyrolysis, reflecting a shift towards more volatile degradation products [57]. Additionally, DGEBA’s limited stability at elevated temperatures is a concern; despite its initially high *T*_g_ values, it is prone to oxidative degradation owing to its aromatic structure [166,167]. Thus, achieving a well-balanced network structure depends on the DGEBA-to-EVO ratio, which ensures sufficient crosslinking, mechanical durability and thermal stability while using the advantages of bio-based alterations.

**Figure 3.**
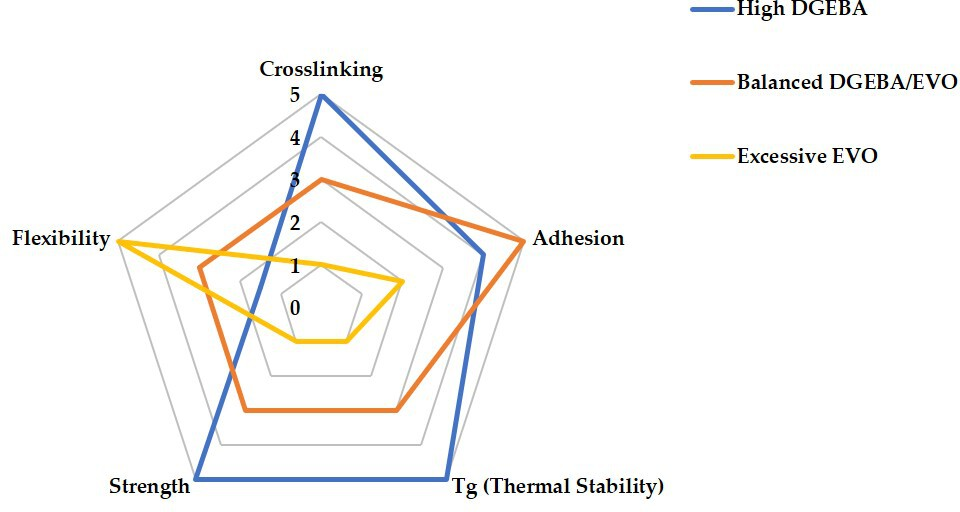
Balance of EVO-to-DGEBA ratio in vitrimer formulation. Note: the radar chart (figure 3) was constructed based on a comparative analysis of tabulated data from table 4, illustrating the effect of EVO-to-DGEBA ratios on key vitrimer properties.

Figure 3 demonstrates how DGEBA/EVO formulations determine the balance between mechanical strength, thermal stability, flexibility and adhesion. High DGEBA content produces a dense, rigid network that enhances strength and heat resistance but limits molecular mobility, reducing flexibility. By contrast, excessive EVO disrupts crosslinking, increasing chain mobility for greater flexibility but weakening intermolecular forces, thereby compromising adhesion and mechanical integrity. For aerospace self-healing materials, networks must both sustain structural loads and autonomously recover from damage; if the network is too rigid, it resists healing, whereas too soft a network may fail to uphold safety standards. Moreover, resin-transfer moulding, widely used in aerospace composite fabrication, requires empirical models that account for temperature and curing-dependent viscosity behaviour to predict flow during mould filling and ensure proper gelation timing [168]. Meanwhile, manufacturing methods like wet filament winding, which are applicable in vitrimer processing, demand longer pot life and controlled viscosity development to enable fibre impregnation and minimize defects. This highlights the critical need to optimize crosslink density to meet diverse processing and performance requirements [169]. Attaining the appropriate stoichiometric equilibrium between bio-based curing agents and epoxy groups is also essential for improving crosslink density, directly influencing material strength and durability [170,171]. Studies report that increasing the epoxy/carboxylic acid ratio raises swelling and lowers gel content, indicating reduced crosslink density, a trend opposite to that observed in tetrafunctional epoxy/dimerized acid systems. Recycling at elevated temperatures promotes catalytic ring-opening polymerization, increasing network density and solvent stability, particularly in formulations with initially lower crosslink density [16]. Using different vegetable oils and fine-tuning curing agent proportions thus enables the development of high-performance bio-based epoxy vitrimers suitable for modern aerospace applications [104].

### 3.3. Influence of epoxidized vegetable oil-to-curing agent on thermal and mechanical performance of vitrimer

The performance of resin-based composite materials is predominantly determined by their molecular structures, which are strongly influenced by curing kinetics parameters such as formulation, temperature and curing time. These parameters govern the final network structure, directly impacting mechanical strength, thermal stability and processability. Effective regulation of the curing process is therefore crucial for optimizing the overall properties of composite materials [172]. Studies have shown that variations in epoxy/acid stoichiometry significantly impact the curing behaviour, crosslink density and ultimately the mechanical and thermal properties of epoxy vitrimers [173]. In bio-based epoxy vitrimers incorporating vegetable oils, this becomes even more significant owing to the lower epoxide functionality of vegetable oil-derived resins, which can result in reduced crosslink density. To counteract this limitation, the curing agent-to-epoxy ratio must be carefully optimized to ensure sufficient network formation while maintaining the inherent flexibility and sustainability benefits of EVO-based epoxy vitrimers. Table 5 further elucidates the impact of stoichiometric ratios of curing agents to EVO on the performance of bio-based vitrimers.

**Table 5.** Influence of EVO-to-curing agent ratio on thermal and mechanical performance.

type of EVO	curing agent	properties of curing agent	curing condition	stoichiometric ratio	thermal properties		mechanical properties				reference
epoxidized hemp oil (EHO) * *M*_W_: 876 g mol^−1^	citric acid (CA)	functions as a crosslinking agent, enhancing network formation	cured with 1 h at 100°C, 1 h at 140°C, 1 h at 160°C and post cured with 5 h at 180°C	EHO/CA :70/30	TGA, residue, %	7.15	sinuous ridges indicate lower crosslinking, promoting ductile flow and energy absorption.				[174]
	tartaric acid (TA)	acts as a curing agent but with limited data on crosslinking efficiency		EHO/TA: 70/30		6.92	smooth, planar cracks suggest high crosslinking, leading to rigidity and poor toughness.				
epoxidized linseed oil (ELSO) EEW: 175 g eq^−1^	citric acid (CA), purity 99.5%,	acts as a multifunctional crosslinker, enhancing network density and self-healing through dynamic bond exchange	cured in 120°C, 24 h	ELSO/CA: 100/20	curing enthalpy (J g^−1^)	≈270	—				[175]
				ELSO/CA: 100/25		≈250					
				ELSO/CA: 100/30		≈230					
				ELSO/CA: 100/33		≈220					
epoxidized linseed oil (ELSO) *M*_W_: 980 g mol^−1^, contains an average of 5.5 epoxy groups per molecule	succinic acid (99.5%),	provides moderate crosslinking and flexibility while maintaining degradability	cured in 180°C during 2 h	ratio, *R* = 0.8	TGA, residue, %	≈ 5	Young ‘s modulus/MPa	78 ± 9	tensile modulus (MPa)	11 ± 2	[176]
	suberic acid (98%),	functions as a flexible crosslinker, balancing mechanical strength and thermal properties				≈ 6		3.9 ± 0.2		0.6 ± 0.1	
	sebacic acid (99%)	acts as a long-chain curing agent, improving thermal stability and flexibility but reducing crosslinking density				≈ 7		4.0 ± 0.3		0.7 ± 0.2	
epoxidized soybean oil (ESO) *ESO, EEW =241 g eq^−1^	2,2’-(ethylenedioxy) -bisethylamine-jeffamine D230	lower physical properties owing to weaker amine reactivity; secondary amine groups at the ends of the polyether chain reduce crosslinking efficiency	cured at 100°C for 24 h and then at 150°C for 48 h	ESO/amine: 1/1.47	—		tensile strength (MPa)	0.11	tensile modulus (MPa)	1.01	[177]
	polyalkyleneamine- jeffamine T403	trifunctional primary amine; secondary carbon amine groups reduce reactivity compared to primary amines		ESO/amine: 1/1.26				1.25		7.5	
	polyalkyleneamine- jeffamine EDR 148	primary amine groups enhance reactivity, providing higher crosslinking efficiency and improved miscibility in solvents		ESO/amine: 1/2.28				1.43		8.0	
	diethylenetriamine (DETA)	contains primary and secondary amine groups; forms a strong polymer matrix with enhanced mechanical properties		ESO/amine: 1/4.10				6.29		231.39	
	triethylenetetramine (TETA)	similar to DETA but with additional secondary amines, improving crosslinking density and network formation		ESO/amine: 1/2.10				8.29		301.60	
	citric acid monohydrate (CA)	forms highly crosslinked networks, enabling stress relaxation, self-healing, and recyclability via transesterification	6 h at 90°C and 12 h at 120°C	ESO/CA: 1 : 0.5			evolution of the relaxation modulus (*G*) at 160°C, f*G*/*G*_0_)		≈0.91		[178]
				ESO/CA: 1 : 0.8					≈0.88		
				ESO/CA: 1 : 1.0					≈0.70		
epoxidized palm oil (EPO) *M*_W_: 1049 g mol^−1^, 0.886 g cm^−3^, OCC = 1.984%	citric acid monohydrate (CA)	forms highly crosslinked networks, enabling stress relaxation, self-healing, and recyclability via transesterification	6 h at 90°C, followed by 12 h at 120°C	EPO/CA: 1 : 0.5	TGA residue, %	≈10	*T*_g_, (°C)		1.42		[179]
				EPO/CA: 1 : 0.8		≈8			4.65		
				EPO/CA: 1 : 1.0		≈7			3.36		
				EPO/CA: 1 : 1.2		≈6			7.87		
				EPO/CA: 1 : 1.5		≈5			8.09		
tung oil (ETO)	citric acid	to attain catalyst free	curing at 140°C for 10 h	ETO/CA: 1 : 0.6	DSC, *T*_g_ (°C)	59.6	adhesion strength (MPa)		≈ 5		[180]
				ETO/CA: 1 : 0.8		72.2			≈ 7.5		
				ETO/CA: 1 : 1.0		85.5			≈ 10		
epoxidized castor oil (ECO)	3-hexahydro−4-methylphtalic anhydride (MHHPA)	acts as an anhydride hardener, enhancing thermal and mechanical stability	140°C for 3 h and 180°C for 2 h	ECO/MHHPA: 1.76/1	TGA residue, %	3.16	solubility in water (100°C, %)		3.21		[181]
				ECO/MHHPA: 1.21/1		3.45			3.68		
				ECO/MHHPA: 0.72/1		1.78			3.06		

Curing agents play a fundamental role in the polymerization of epoxy monomers, facilitating crosslink formation that reinforces structural integrity [182]. The curing agent-to-epoxy ratio directly influences mechanical strength and thermal stability, with deviations from an optimal ratio leading to performance deterioration [172]. Excessive curing agent promotes over-crosslinking [183], increasing brittleness [184], induced unwanted creep deformation [185] and restricts molecular mobility [153], making the material more susceptible to fractures under stress. Several studies have demonstrated that excessive crosslinking reduces tensile strength and elongation at break by disrupting the resin structure and causing molecular chain breakage. Increased curing agent content further extends stress relaxation time without enhancing mechanical properties, as steric hindrance limits effective crosslinking, weakening vitrimer integrity [186,187]. Conversely, an insufficient amount of curing agent results incomplete curing of the thermoset [188], leading to lower mechanical strength and reduced chemical resistance owing to unreacted epoxy groups remaining in the network [189,190]. Thus, establishing an optimal curing agent-to-epoxy ratio is crucial for maintaining crosslinking efficiency while preserving the durability and reprocessability of bio-based vitrimer formulations. Achieving an appropriate ratio ensures sufficient crosslinking for structural integrity while retaining the dynamic adaptability of the network, which is essential for self-healing and recyclability.

By varying crosslinking density, network rigidity and dynamic bond formation, the type and ratio of the curing agent significantly affect vitrimer characteristics. As shown in table 5, CA and tartaric acid (TA) in epoxidized hemp oil (EHO) vitrimers exhibit contrasting effects which CA imparts flexibility, whereas TA creates a highly rigid structure owing to its stronger crosslinking [191]. The contrasting effects of CA and TA on the properties of EHO vitrimers can be attributed to their carboxyl (COOH) groups. One molecule of CA, containing three COOH groups, forms flexible crosslinks through esterification reactions, promoting self-healing properties in the vitrimer network [192,193]. By contrast, TA, with two COOH groups, creates a denser, more rigid network owing to stronger crosslinking, enhancing mechanical strength but reducing self-healing potential. The curing ratio determines not only crosslinking but also self-healing efficiency by transesterification processes for ESO and EPO cured with CA. While too high CA generates rigid domains that impede chain mobility and limit dynamic bond interchange, insufficient CA results in an incomplete polymer network with worse mechanical characteristics and lower thermal stability [178]. Higher CA concentrations also encourage porosity, therefore weakening the vitrimer matrix and raising brittleness resulting from network discontinuities [194]. This implies that to sustain stress relaxation and self-repair capacity, an optimum CA ratio balances mechanical integrity with polymer mobility. Whereas long-chain acids like sebacic acid introduce flexibility by lowering crosslink density, short-chain acids like CA and succinic acid create densely crosslinked but stiff networks [195,196]. Notably, there is an optimal curing agent ratio for each vitrimer formulation, beyond which mechanical performance declines. In the EPO/CA formulation, a 1 : 1.5 ratio achieves the highest *T*_g_, but excessive CA leads to plasticization, reducing thermal stability [179].

Beyond crosslinking, the type of curing agent used greatly affects the vitrimer’s ultimate qualities. ESO-based vitrimer using polyamine curing agents, particularly diethylenetriamine (DETA) and triethylenetetramine (TETA), enhance mechanical properties by promoting a tightly packed polymer network. However, excessive TETA induces internal stresses and brittleness owing to constrained molecular mobility [197]. Likewise, in ESO-based vitrimers, excessive jeffamine content results in over-crosslinking and embrittlement [177]. When ESO is cured with polyamine-based curing agents, the curing ratio influences more than just crosslinking. However, excessive use of the polyamine curing agents can lead to increased network density, enhancing mechanical properties but also causing curing shrinkage. This shrinkage may lead to microvoid formation, affecting toughness and fracture resistance [198,199]. Meanwhile, secondary amines, such as jeffamine derivatives, exhibit lower reactivity, resulting in slower curing kinetics and the formation of a more heterogeneous network with localized soft and hard phases [200,201].

Similarly, highly reactive primary amines like DETA and TETA promote stiffness but may compromise impact resistance, while multifunctional amines such as jeffamine T403 improve toughness [202,203]. In line with advancing high-performance vitrimer systems, carbon fibre-reinforced composites have also been investigated for aerospace applications. A recent study transformed a space-grade epoxy thermoset matrix into a high *T*_g_ vitrimer (≈ 200°C) by incorporating disulphide exchange chemistry using 4-aminophenyl disulfide. Notably, off-stoichiometric formulations (epoxy/amine = 1/1.2) retained a high *T*_g_ of 175°C while enhancing reprocessability and maintaining excellent mechanical and outgassing properties [204]. Meanwhile, according to the study by Asempour & Marić [205], bio-derived vitrimers synthesized from terpene-based β-myrcene crosslinked with difunctional or trifunctional amines exhibited tunable mechanical and rheological properties depending on crosslinker type and density. These vitrimers preserved dynamic bond exchange behaviour over multiple reprocessing cycles, with dual static and dynamic cross-links improving creep resistance and shape memory effects. All these studies highlight that the balance between curing agent type, ratio and network formation is essential in achieving the desired vitrimer properties.

**Figure 4.**
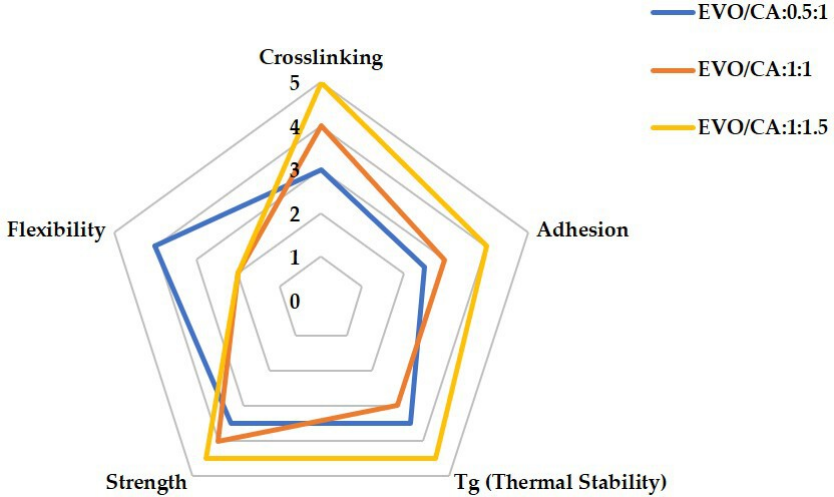
Effect of EVO-to-curing agent ratio in vitrimer formulation. Note: the radar chart (figure 4) was constructed based on a comparative analysis of tabulated data from table 5, illustrating the effect of EVO-to-curing agent ratios on key vitrimer properties.

Figure 4 shows the effects on important thermal and mechanical parameters of many EVO/curing agent ratios (0.5 : 1, 1 : 1 and 1.5 : 1). It shows great flexibility but reduced crosslinking, adhesion, and thermal stability, indicating poor structural integrity. The 0.5 : 1 ratio (blue) suggests a best trade-off between flexibility and mechanical strength, the 1 : 1 ratio (orange) offers a balanced performance across all attributes. Though at the expense of less flexibility, the 1.5 : 1 ratio (yellow) gets the best values in adhesion, crosslinking, strength and heat stability. This study implies that the most effective balance of mechanical and thermal characteristics is given by the 1 : 1 EVO/curing agent ratio.

### 3.4. Effect of epoxidized vegetable oil-to-catalyst ratio on thermal and mechanical performance

In epoxy-based vitrimers, catalysts play a critical role in accelerating transesterification, facilitating efficient polymer chain exchange and improving reprocessability. Without sufficient catalyst loading, the reaction rate slows, limiting the vitrimer’s ability to self-heal and undergo multiple recycling cycles [206]. To enhance dynamic bond exchange, various specific catalysts are employed, including Lewis and Brønsted acids, zinc salts, triphenylphosphine and tertiary amines [207]. These catalysts lower the activation energy required for transesterification, promoting faster network rearrangement and improving vitrimer adaptability. However, their effectiveness depends on factors such as thermal stability, compatibility with the polymer matrix and interactions with the crosslinked structure [208]. A key challenge in catalyst-based vitrimers is the potential instability of certain catalysts at high temperatures, which can lead to leaching during reprocessing, ultimately reducing long-term recyclability and mechanical performance [209,210]. Another challenge is that catalysts may necessitate encapsulation to preserve their activity, hence complicating the construction of a self-healing mechanism [211]. The inclusion of catalysts in the coating matrix is crucial for preserving vitrimer functionality; nevertheless, their addition as a secondary phase may result in considerable variability within the matrix [212].

Table 6 shows that catalyst loading critically impacts vitrimer performance, emphasizing the need to identify an optimal concentration. For instance, Li *et al.* [214] demonstrated that increasing Zinc acetate, Zn(OAc)₂ content up to 0.5% raises tensile strength to a maximum of 42 MPa by enhancing stress transfer and crosslinking efficiency. Beyond 0.5%, additional Zn(OAc)₂ decreases tensile strength owing to disruption of crosslink density and network uniformity [218]. Similarly, research by Cincilio *et al.* [219] showed that increasing zinc octoate (Zn(Oct)₂) enhances stress relaxation at elevated temperatures by accelerating transesterification. This process facilitates the reorganization of the polymer network, effectively relieving deformation-induced stress because of a lower crosslinking density. However, beyond this threshold, additional catalyst loading (1% and 2%) resulted in a decline in mechanical performance. A similar trend was observed in [187], where an increase in triazabicyclodecene (TBD) from 5% to 10% enhanced mechanical properties, but a further increase to 15% reduced the tensile strength owing to excessive plasticization. These results suggest that while catalysts promote transesterification and stress relaxation, excessive amounts may disrupt the crosslinked network, leading to structural weaknesses [209,220]. These contrasting findings highlight the complex role of catalyst concentration in epoxy-based vitrimer performance, where an optimal balance is crucial to achieving both efficient stress relaxation and mechanical durability.

**Table 6.** The effect of EVO-to-catalysts ratio in vitrimer performance.

type of epoxy	type of EVO	curing agent	catalysts	catalyst concentration	thermal properties				mechanical properties				reference
DGEBA	—	sebacic acid, 99%	1,5,7-triazabicyclo [4.4.0] dec−5-ene	blank	—				Young’s modulus (GPa)		1.7		[213]
				1 mo%	*T*_g_ (°C)	≈ 35	tv, topology freezing °C	≈ 250					
				5 mol%		≈35		≈ 200			1.1		
DGEBA 0.44/100 mol g^−1^	—	dodecanedioic acid	zinc acetate Zn (OAc)_2_	blank	DSC, *T*_g_ (°C)		≈ 30.1		tensile (MPa)	18 ± 1	Young’s modulus (MPa)	156 ± 12	[214]
				0.25%			≈ 48.9			37 ± 2		267 ± 16	
				0.5%			≈ 45.9			42 ± 2		307 ± 11	
				1%			≈ 45.8			39 ± 2		270 ± 10	
				2%			≈ 45.3			38 ± 2		257 ± 12	
DGEBA EEW: 174		sebacic acid, 97%	1,5,7-triazabicylo [4.4.0] dec−5-ene (TBD), 97%	5%	T5% weight loss (TGA, °C)		≈360−370		tensile strength (MPa)		≈40		[187]
				10%			≈320−340				≈45		
				15%			≈310−330				≈35		
DGEBA, EEW: 184−194 g		1,5,7-triazabicyclo [4.4.0] dec−5-ene	graphene	blank	T5% weight loss (TGA, °C)		345		tensile (MPa)	12.0 ± 0.8	Young’s modulus (MPa)	565.9 ± 10.1	[215]
				0.1%			344			13.4 ± 1.2		767.3 ± 8.5	
				0.5%			332			14.4 ± 1.0		797.5 ± 7.9	
				1%			348			22.9 ± 1.7		1232 ± 23.5	
				3%			355			17.4 ± 0.6		1022 ± 14.3	
—	ESO	1,5,7-triazabicyclo [4.4.0] dec−5-ene, 98%	natural glycyrrhizic acid	0.4%	T5% weight loss (TGA, °C)		315						[216]
				0.5%			328						
				0.6%			314						
		fumaropimaric acid (FPA)	zinc acetylacetonate (Zn (ACAC)_2_,	5:0.19			sample (5 : 0.19) had a higher *T*_d_ 5% than the other two		tensile strength (MPa)			≈ 16	[207]
				5:0.15								≈9	
				5:0.11								≈6	
—	ELSO	2,2′-dithiodibenzoic acid (DTBA)	—	—	T5% weight loss (TGA, °C)		230		—				[163]
			imidazole (IM)				275						
			1-MI – 1-methylimidazole				265						
			2-MI – 2-methylimidazole				250						
			1,2-dimethylimidazole (1,2-DMI)				264						
			2-ethyl−4-methylimidazole (2E4MI)				260						
			N, N-dimethylbenzylamine (DMB)				255						
			dimethylaminopyridine (DMAP)				245						
			2,4,6-tris-(dimethylaminomethyl)phenol (DMP-30)				245						
			1-methylpiperazine (1-MP)				250						
			1,5,7- triazabicyclo [4.4.0] dec−5-ene,				260						
	epoxidized jatropha oil	tetramethylolpropane triacrylate	nano zinc oxide (ZnO)	blank	TGA (residue, %)		≈ 1−2		pull-off adhesion (psi)		45.7 ± 1.7		[217]
				1 wt%			≈ 5−7				85.3 ± 6.3		
				3 wt%			≈ 10−12				± 5.2		
				5 wt%			≈ 15−18				133.0 ± 5.7		
				7 wt%			≈ 20−22				77.3 ± 2.1		
				9 wt%			≈ 25−28				66.0 ± 2.4		
—	epoxidized canola oil (ECNO) *M*_W_: 865 g mol^−1^, EEW: 229.75 g eq^−1^	racemic lactic acid (LA), 84.5−85.5%	—	blank	DSC, *T*_g_ (°C)		≈ −15.2						[208]
			zinc acetate (ZnAc), purity > 98%	1%			≈ −16.3						
				2%			≈ −18.65						
			ZnAl-layered double hydroxide (ZnAl), Zn/Al molar ratio = 4.0	1%			≈ −21.15						
				2%			≈ −23.1						

TGA = Thermogravimetric Analysis

In addition to EVO-to-catalyst ratio, the nature of the catalyst itself significantly influences the thermal and mechanical properties of vitrimer systems. For example, Leibler and coworkers reported that epoxy-based transesterification vitrimers require high catalyst loadings and elevated temperatures to reach sufficient exchange rates. Dibutyltin dilaurate (DBTL), a Lewis acid catalyst, was also shown to lower activation energy via carbonyl activation, allowing faster reactions at lower temperatures. However, DBTL also exhibited inhibitory effects, depending on network composition. Similarly, the use of TBD increased activation energy, shifting the mechanism to zwitterionic addition/elimination pathways. These findings underscore that catalyst identity and loading can profoundly influence vitrimer dynamics, affecting reprocessability, dimensional stability and overall performance [221].

Over the past few years, carbon-derived reinforcing agents such as graphene, graphene oxide, reduced graphene oxide, activated carbon and carbon nanotubes (CNTs) have gained significant attention in biocomposite materials because of their ability to enhance mechanical, thermal and electrical properties [206,222]. A report by Yang *et al.* [223] demonstrated that graphene, when used as a catalyst in epoxy-based vitrimers, significantly improved mechanical performance by promoting efficient crosslinking, leading to greater strength and durability. Similarly, CNTs have been explored as high-performance fillers for vitrimers owing to their high aspect ratio, large surface area and exceptional thermal, chemical and electrical properties. Their dispersion in vitrimer formulation not only enhances mechanical and thermal stability but also introduces photo-thermal effects, further expanding the material’s functionality. Also, this nanomaterial holds future application in lightweight structural components, conductive materials for aircraft systems, electromagnetic interference shielding, advanced sensors, energy storage systems, and nanocoatings for aerospace and other advanced industries [224]. While poor dispersion of CNTs is often blamed for property degradation, evidence shows that CNT agglomerates create severe matrix stress concentrations, with their intensity depending on agglomerate density, size, and degree of agglomeration. Even partial CNT clustering can provoke significant stress concentrations that accelerate damage onset in the composite [225]. To promote sustainability, recent studies have investigated biomass-derived carbon as an alternative filler. Krishnakumar *et al.* [226] used activated carbon derived from sugarcane bagasse in DGEBA-based vitrimers, demonstrating that its high surface area facilitates progressive chain exchanges, thereby improving the vitrimer’s dynamic adaptability and recyclability. However, excessive loading can also lead to agglomeration of activated carbon particles, which trap gases, increase porosity and fail to fill microvoids which ultimately generate stress concentrations that weaken the structure and reduce tensile strength, with even partial clustering promoting earlier damage initiation [227,228]. To mitigate such dispersion issues, ultrasonication is often employed to break agglomerates and achieve a more uniform distribution of nanoparticles within the vitrimer matrix [229]. Looking ahead, EVO-based epoxy formulation holds promise as a sustainable alternative to DGEBA, offering the potential to incorporate bio-based-activated carbon and catalysts for the development of environmentally friendly vitrimer materials with enhanced performance and circularity.

As shown in table 6, the use of EVO such as soybean, linseed and canola oils in vitrimer formulations has gained increasing attention owing to their renewable nature and tunable properties. The selection and ratio of catalysts play a crucial role in determining the thermal and mechanical performance of these materials. For instance, ESO combined with glycyrrhizic acid achieved a *T*_5%_ of 328°C, while fumaropimaric acid with Zn(acac)₂ enhanced mechanical strength to approximately 16 MPa, demonstrating the influence of catalyst selection on thermal stability and mechanical properties [207,230]. Similarly, imidazole-based catalysts in ELSO improved *T*_5%_ values up to 275°C, while the incorporation of Zn-based fillers in epoxidized canola oil effectively adjusted *T*_g_, influencing the material’s flexibility and adaptability [163,208]. These findings highlight the potential of EVO-based vitrimers as customizable and efficient alternatives to conventional resins, where catalyst optimization plays a key role in enhancing performance and sustainability.

Furthermore, we discovered that the EVO-based vitrimers employ an entirely different approach compared to other materials, relying less on external catalysts because of their unique chemical structure. Precursors such as EPO and epoxidized jatropha oil contain hydroxyl and carboxyl functional groups, which naturally facilitate transesterification reactions without the need for additional catalysts [223,231]. This built-in reactivity not only simplifies the vitrimer formulation but also reduces the risk of catalyst-related drawbacks, such as plasticization and leaching. Moreover, many formulations also incorporate bio-based acids, such as citric or tartaric acid, which serve as both curing agents and self-catalysts, facilitating bond rearrangement without the need for metal-based catalysts [231,232]. However, the absence of catalysts can sometimes limit the efficiency of dynamic bond exchange, potentially affecting long-term durability and performance under repeated reprocessing cycles [233]. Although EVO-based vitrimers largely rely on intrinsic reactivity, catalysts may still remain essential for optimizing their properties.

Our review addresses a key research gap by illustrating that EVO-based vitrimers necessitate considerably lower catalyst concentrations compared to epoxy-based formulation. EVOs, in contrast to traditional epoxies, demonstrate enhanced functionality and reactivity of aliphatic chains, facilitating effective bond exchange with reduced catalyst requirements. Epoxy-based vitrimers generally necessitate 0.5−5% catalyst, whereas EVO formulations get the same or enhanced performance with merely 0.4−1%. This diminished reliance on catalysts not only reduces expenses but also mitigates the possibility of leaching, hence enhancing long-term material stability. Our findings advocate for the construction of more sustainable, high-performance vitrimers by optimizing the stoichiometric ratio between catalysts and EVO precursors, hence minimizing catalyst use.

**Figure 5.**
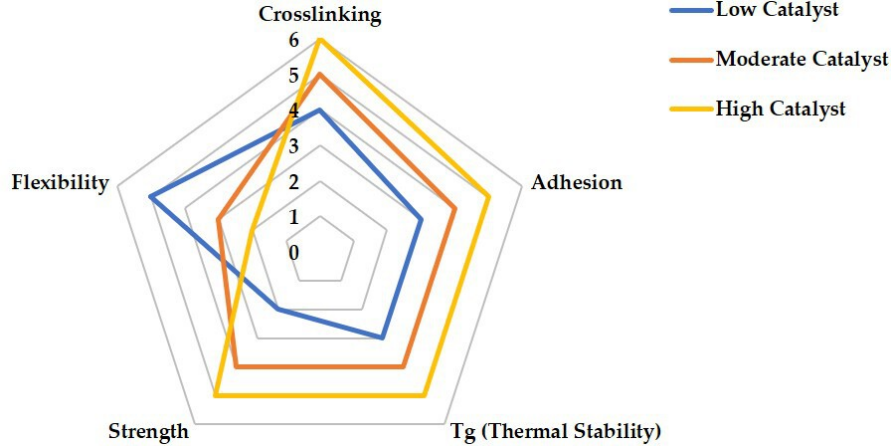
Balance of EVO-to-catalyst ratio in vitrimer formulation. Note: the radar chart (figure 5) was constructed based on a comparative analysis of tabulated data from table 6, illustrating the effect of EVO-to-catalyst ratios on key vitrimer properties.

Figure 5 demonstrates the significant impact of catalyst concentration on the vitrimer formulation. Increased catalyst concentrations expedite network formation, improving crosslink density, adhesion and thermal stability. Excessive catalyst loading (yellow) creates internal stresses, resulting in embrittlement, microcrack development and diminished impact resistance, while also heightening the danger of catalyst leaching. Unreacted or weakly attached catalyst molecules may migrate to the surface over time, undermining long-term durability and potentially leading to interfacial adhesion failure. The leaching effect diminishes mechanical performance and heightens worries about environmental stability, especially in coatings subjected to moisture or chemical interactions. The economic ramifications of catalyst optimization extend beyond just physical performance. The excessive use of catalysts elevates production costs without guaranteeing corresponding performance advantages, rendering the formulation less economically viable. Conversely, inadequate catalyst concentrations (blue) result in incomplete curing, diminished intermolecular connections and lowered load-bearing capability, necessitating further processing or material alterations to address these shortcomings. Attaining the optimal catalyst concentration provides a regulated reaction rate, harmonizing polymer network development, mechanical strength and cost-effectiveness, while reducing waste and material deterioration over time. Optimizing catalyst loading is vital for enhancing structural performance and is critical for developing a sustainable, high-performance epoxy formulation that satisfies economic and environmental criteria.

## 4. Precise calculation in vitrimer formulation

In vitrimer material formulation, achieving the correct stoichiometric balance is crucial for optimizing crosslinking efficiency, mechanical properties and dynamic adaptability. The formulation is governed by accurate determination of the appropriate ratios, which influence the vitrimer’s thermal stability, reprocessability and self-healing efficiency:

(i) DGEBA-to-EVO ratio;
(ii) EVO-to-curing agent ratio; and
(iii) EVO-to-catalyst ratio.

### 4.1. Stoichiometric calculations for diglycidyl ether of bisphenol-A/epoxidized vegetable oil-based vitrimer

The DGEBA-to-EVO ratio is determined based on two key parameters: epoxy equivalent weight (EEW) and oxirane oxygen content (OOC%). These two key parameters are critical parameters in epoxy resin formulations, influencing crosslinking density, viscosity, reaction kinetics, and thermal stability. Understanding their effects is essential for tailoring vitrimer properties to specific applications.

#### 4.1.1. Epoxy equivalent weight

EEW is a fundamental parameter in epoxy resin formulation, representing the mass of epoxy resin (in grams) required to provide one mole-equivalent of reactive epoxy groups. Expressed in g eq^−1^, EEW is essential for accurate stoichiometric calculations, especially in determining the correct ratio of curing agents. While it is related to the resin’s structure, EEW does not represent the overall molecular weight of the resin; instead, it reflects the specific weight necessary to supply one equivalent of epoxy functionality. For example, EEW is approximately one-half the average molecular weight of a diepoxy resin or one-third for a triepoxy resin, depending on the number of epoxy groups present per molecule [234]. This parameter is crucial for ensuring the correct stoichiometric balance when combining epoxy resins with curing agents or other reactive components [235]. The formula for EEW is: [236]


$$EEW\;=\;\frac{molecular\;weight\;of\;epoxide-containing\;resin\;}{number\;of\;epoxide\;group\;per\;molecule}.$$

For typical epoxides:

— DGEBA EEW≈ 170−190 g eq^−1^ (depending on polymerization degree); and
— EVO (epoxidized soybean-based) EEW ≈ 250−300 g eq^−1^ (varies with oil structure and epoxidation level).

If DGEBA EEW = 180 g eq^−1^ and EVO EEW = 270 g eq^−1^, then the stoichiometric balance is:


$$\frac{mass\;of\;DGEBA}{mass\;of\;EVO}\;=\frac{270}{180}\;=\;1.5.$$

If a 50 : 50 weight ratio is used (e.g. 100 g of DGEBA and 100 g of EVO), the vitrimer will have a lower overall epoxy functionality because of EVO’s higher EEW (270 g eq^−1^) compared to DGEBA (180 g eq^−1^). This imbalance leads to a reduced crosslink density, potentially affecting the mechanical strength, curing efficiency, and final properties of the epoxy network. Therefore, adjusting the ratio based on EEW is crucial for maintaining the desired performance and structural integrity of the formulation.

#### 4.1.2. Oxirane oxygen content

Likewise, OOC% indicates the availability of reactive epoxy groups for crosslinking by measuring the percentage of oxirane (epoxide) oxygen in the resin. Research indicates that oxygen impacts the main chain of the epoxy resin by establishing carbon–oxygen double bonds, therefore influencing the thermal stability of the epoxy resin and hence its features of thermal breakdown [237]. More reactive sites indicated by a higher OOC% can hasten the curing process. For instance, it has been noted that adding ESO which raises the oxirane content—changes the kinetics of curing in epoxy formulation [127].

OOC% determines the epoxide group availability and is given by:


$$OOC\%=\frac{molecular\;weight\;of\;oxirane\;oxygen\;\times \;number\;of\;epoxide\;groups}{molecular\;weight\;of\;epoxidized\;resin}\times 100.$$

For typical epoxides:

— DGEBAOOC ≈ 8.5% [238]; and
— EVO (epoxidized soybean-based) OOC ≈ 5.5% [239].

If DGEBA OOC = 8.5% and EVO OOC = 5.5%, then the stoichiometric balance is calculated as:


$$\frac{mass\;of\;DGEBA}{mass\;of\;EVO}\;=\;\frac{5.5}{8.5}=0.65.$$

If a 50 : 50 weight ratio is used (e.g. 100 g of DGEBA and 100 g of EVO), the formulation will have an imbalanced OOC% owing to EVO’s lower OOC (7.5%) compared to DGEBA (8.5%). This imbalance results in an excess of DGEBA relative to EVO, which could lead to over crosslinking or incomplete curing. The reason more EVO is required is that EVO has a lower OOC%, necessitating the use of a higher proportion of DGEBA to ensure an equivalent concentration of reactive epoxide groups. Therefore, adjusting the ratio based on OOC% is essential for achieving optimal curing efficiency and maintaining the desired performance and structural integrity of the final vitrimer formulation.

### 4.2. Epoxide-to-carboxyl stoichiometry in epoxidized vegetable oil-curing agent formulation

The reaction between epoxide groups in EVO and functional groups in curing agents, such as carboxyl (-COOH) or amine (-NH), is crucial for achieving efficient crosslinking and curing [240]. This directly impacts the final material properties, including mechanical strength, chemical resistance and thermal stability [16]. Table 5 highlights the importance of maintaining a proper stoichiometric balance to ensure optimal curing and material performance. The stoichiometry in EVO-curing agent formulation is determined using two key parameters: EEW of EVO and the equivalent weight of the curing agent, which varies based on whether the curing agent is carboxyl-based amine equivalent weight (AEW) or amine-based (amine hydrogen equivalent weight (AHEW)):

(i) EEW: this is the weight of EVO required to provide one mole of epoxide groups. It determines the amount of EVO needed for the reaction with curing agents (as stated in §4.1.1); and
(ii)curing agent equivalent weight: depending on the type of curing agent, the equivalent weight is calculated as follows.

#### 4.2.1. Stoichiometry for carboxyl-based curing agents

AEW for carboxyl-based curing agents:


$$AEW=\frac{molecular\;weight\left(MW\right)}{number\;of\;COOH\;groups}.$$

Citric acid (CA), three COOH groups:


$$AEW=\frac{192}{3}=64\;g\;eq^{-1}.$$

The stoichiometric ratio is determined by the ratio of the EEW of EVO to the AEW of the curing agent:


$$\frac{mass\;of\;EVO}{mass\;of\;curing\;agent}=\frac{EEW\;of\;EVO}{AEW\;of\;curing\;agent}.$$

For instance, if EVO has an EEW of 270 g eq^−1^ and CA has an AEW of 64 g eq^−1^, the required mass ratio is:


$$\;\frac{mass\;of\;EVO}{mass\;of\;curing\;agent}=\frac{270}{64}\approx 4.22.$$

If a 50 : 50 weight ratio of EVO to curing agent is used, an imbalance may occur because of the excess epoxide groups from EVO relative to the available carboxyl groups. This could lead to incomplete curing or excessive crosslinking, affecting the material’s flexibility, hardness and overall performance. Because EVO has a lower reactive group concentration, adjusting the ratio based on functional group equivalence is essential to ensure efficient curing and maintain the desired vitrimer properties.

#### 4.2.2. Stoichiometry for amine-based curing agents

**Figure 6.**
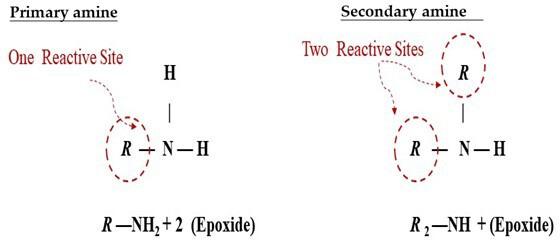
Comparison of primary and secondary amines based on their reactive sites towards epoxide groups.

Amines require special attention owing to differing numbers of reactive hydrogens per nitrogen. As shown in figure 6, primary amines (–NH₂) contain two reactive hydrogens per nitrogen, allowing each to open two epoxide rings, while secondary amines (–NHR) contain one reactive hydrogen and react more slowly. Tertiary amines lack reactive hydrogens and do not contribute to crosslinking [241].

The AHEW is calculated by:

AHEW for amine-based curing agents:


$$AHEW=\frac{molecular\;weight\left(MW\right)}{number\;of\;active\;hydrogen\;atoms}.$$

The AHEW is calculated by:


$$AHEW=\frac{molecular\;weight\left(MW\right)}{2\times N_{prim}\;\;+\;1\times N_{sec}}.$$

where *N*_prim_ and *N*_sec_ are the numbers of primary and secondary amine nitrogens, respectively. For example, a curing agent with *M*_W_ = 120 g mol^−1^, 1 primary, and 1 secondary amine nitrogen has:


$$AHEW=\frac{molecular\;weight\left(MW\right)}{2\times 1\;\;+\;1\times 1}\;=\;\frac{120}{3}=\;40\;g\;eq^{-1}.$$

The stoichiometric mass ratio of EVO to this curing agent is:


$$\begin{array}{rl} \frac{mass\;of\;EVO}{mass\;of\;curing\;agent} & =\frac{EEW}{AHEW}\\ & =\frac{270}{40}\approx 6.75. \end{array}$$

This indicates that approximately 6.75 parts by mass of EVO resin are required for each part of the amine-based curing agent to achieve the correct reactive group balance. Failure to maintain this stoichiometric ratio can lead to either an excess of unreacted epoxide groups or a shortage of available amine hydrogen, resulting in incomplete curing, reduced crosslink density and inferior vitrimer performance. Therefore, careful calculation of AHEW is essential to ensure accurate formulation and optimal vitrimer network formation.

##### 4.2.2.1 Additional considerations

This stoichiometric calculation assumes all amines are equally reactive proportional to their number of active hydrogens. Real systems may exhibit kinetic differences, but this approach provides a stoichiometrically sound basis for formulation. Accurate stoichiometric balance is essential to avoid incomplete curing, residual unreacted groups or excessive crosslinking, all of which impact the vitrimer’s mechanical and thermal properties.

### 4.3. Epoxidized vegetable oil : catalyst ratio (weight-based calculation for dynamic bond exchange)

Generally, catalyst loading in biocomposites typically ranges from 1% to 5% by weight, balancing performance and cost efficiency [228,242,243]. Catalysts facilitate transesterification reactions, enabling dynamic bond exchange and controlling vitrimer properties. The amount of catalyst required is determined using the following formula:


$$m{\;}_{cat}=\left(\frac{catalyst\;loading\;\left(\%\right)}{100}\right)\;\times \;m{\;}_{EVO}.$$

Through precise stoichiometric calculations, vitrimer formulations can be optimized to achieve a balance of mechanical strength, crosslinking efficiency and dynamic adaptability. Ensuring the correct DGEBA : EVO, EVO : curing agent and EVO : catalyst ratios is fundamental to developing high-performance, self-healing and reprocessable vitrimer networks.

### 4.4. Impact of non-ideal stoichiometry on vitrimer network dynamics

Research on vitrimers has advanced through precise formulation calculations, yet the control of bond exchange kinetics under non-ideal conditions remains essential. Konuray *et al*. [244] has shown that the relaxation behaviour of step-growth polymerized thermosets can be adjusted by modifying the network topology and introducing multiple dynamic bond exchange reactions with distinct kinetics. Among these systems, thiol-click thermosets have attracted considerable attention for their vitrimer-like behaviour. Although thiol-click reactions are typically viewed as ideal stepwise crosslinking processes, deviations have been observed in off-stoichiometric thiol-epoxy systems. These are often linked to intramolecular cyclization, a behaviour also seen in epoxy-amine networks where flexible chains favour ring formation during curing. This non-ideal behaviour may lead to delayed gelation, changes in the critical gelation ratio, decreased crosslink density, and a higher soluble fraction [245–248]. While ideal stepwise polymerization assumes a stoichiometric balance and the absence of side reactions, real systems frequently deviate from this assumption [249]. Overcoming these limitations is important for improving vitrimer properties and requires deeper insights into bond exchange kinetics, defect formation and advanced modelling approaches.

## 5. Conclusion and future perspectives

This review research explores both full and substantial replacement of DGEBA with EVO while identifying the advantages and limitations of both formulations, offering a balanced perspective compared to conventional approaches that fully substitute DGEBA. This study formulates a framework for tailoring vitrimer formulations to address individual application requirements by exploiting DGEBA’s higher crosslinking density and inherent rigidity, in conjunction with EVO’s sustainability and enhanced reprocessability. Understanding these differences highlights areas where EVO requires improvement and enables strategic modifications to enhance its performance while retaining key advantages of DGEBA. These findings serve as a foundation for refining bio-based vitrimer formulations to ensure both durability and functionality. A key aspect of this research is the role of curing agents in vitrimer performance. Variations in curing agent functionality, particularly the presence of carboxyl (-COOH) and amine (-NH) groups, significantly impact reaction kinetics, crosslinking efficiency and overall material properties. This study demonstrates that amine-based curing agents promote higher crosslinking density and improved mechanical strength, whereas carboxyl-based curing agents contribute to greater flexibility and enhanced self-healing capabilities. By evaluating these functional group variations, this study establishes a framework for tailoring vitrimer formulations to meet specific application needs. Furthermore, this study reveals that EVO-based vitrimer formulations require significantly lower catalyst concentrations compared to epoxy-based counterparts. While conventional epoxy-based vitrimers typically demand 0.5−5% catalyst loading, EVO-based formulations achieve comparable or superior performance with just 0.4−1%. This reduced catalyst demand not only minimizes potential leaching but also enhances catalyst efficiency in facilitating dynamic bond exchange, improving reprocessability without compromising mechanical integrity. Such optimization strengthens the viability of EVO as a sustainable alternative to petroleum-derived formulations while maintaining high-performance characteristics. Strengthening stoichiometric precision through fundamental calculations, as presented in this study, serves as a cornerstone for future vitrimer research and development, ensuring precise control over material properties and formulation accuracy. Future research should concentrate on enhancing the interaction between EVO and curing chemicals, while also optimizing catalyst efficiency using sustainable alternatives such as cellulose nanofibres or activated carbon. Integrating bio-based catalysts into EVO vitrimer formulations may enhance mechanical performance and environmental sustainability in future developments.

## Data Availability

This article has no additional data.
